# RISK EVALUATION IN THE LOW-DOSE RANGE CT FOR RADIATION-EXPOSED CHILDREN, BASED ON DNA DAMAGE

**DOI:** 10.1093/rpd/ncz195

**Published:** 2019-10-29

**Authors:** Lenka Jánošíková, Martina Juričeková, Martina Horváthová, Denisa Nikodemová, Andrej Klepanec, Dušan Šalát

**Affiliations:** 1 Institute of Physiotherapy, Balneology and Medical Rehabilitation, University of Ss. Cyril and Methodius in Trnava, Trnava, Slovakia; 2 Faculty of Health Care and Social Work, University of Trnava in Trnava, Trnava, Slovakia; 3 Slovak Medical University in Bratislava, Slovakia

## Abstract

One of the most common usages of radiation in current medical diagnosis is computed tomography (CT) using X-rays. The potential health risk of CT scans has been discussed in various studies to determine whether low-dose radiation from CT could enhance the chromosome aberration yields in pediatric patients and increase their risk of carcinogenesis. For this reason, it is of great interest to study the effects of low-dose radiation. The induction of DNA damage by a CT scan examination has been demonstrated in several reports by the γ-H2AX assay, the micronuclei assay and dicentrics measurements. However, the results of most studies showed limitations. On the other hand, epidemiological studies give contradictory results for post-natal radiation exposure in the low-dose range, so it is still difficult to draw conclusions about the effects of CT examinations and risk of carcinogenesis. This article provides an overview of previously published data and summarizes the current state of knowledge.

## INTRODUCTION

Diagnostic radiation is an indispensable tool of modern medicine. Among the uses of X-rays in diagnosis, computed tomography (CT) has been established as one of the most informative diagnostic radiology examinations. However, the growing use of CT procedures on children raises concern over the long-term risk of cancer development associated with medical radiologic diagnostics^(^^1)^, because children are more radiosensitive than adults^(^^2)^. They are more radiosensitive due to their growing bodies, rapid cell proliferation allowing repair of DNA damage, expansion of altered cell clones and longer lifespans, which provide a larger window for development of radiation-related cancers^(^^3–5)^. It is urgently needed to find out the underlying mechanism of radiosensitivity.

### Epidemiological studies

The epidemiological studies give contradictory results for postnatal radiation exposure in the low-dose range^(^^6)^. Some investigators reported no association with an increase of future cancer risk from CT scans^(^^7^^,^^8)^. Other studies observe the association of childhood cancer risk with CT scan examination^(^^9–11)^. Two cohort studies from Australia and from Great Britain reported a significantly increased leukemia and brain cancer risk after receiving CT scans during childhood^(^^10^^,^^9)^. Another cohort study from Taiwan reported an association with an increase of benign brain tumor after pediatric head CT examination^(^^12)^. However, their findings were criticized because epidemiological patterns reported in the CT studies were inconsistent with the world’s literature. For example, in the UK study, teenagers had a higher risk of a brain tumor than young children; in the Australian study, cancers not previously linked to radiation were significantly elevated; and in the Taiwanese study, the risk of benign tumors decreased with age at the time of CT examination. In all studies, solid tumors appeared much earlier than previously reported^(^^13)^. Cancer risk estimates for CT scans are typically extrapolated from models, therefore, new approaches measuring actual DNA damage on the molecular level are clearly needed for improved risk estimations^(^^14)^.

### DSB after CT

Radiation-induced DNA damage originates from both reactive oxygen species created along the radiation track and direct electron interaction with DNA. The damage to DNA typically causes single-strand breaks, double-strand breaks (DSBs) and crosslinks^(^^15)^; among these lesions, DSBs are considered to be the major actor responsible for cell death. If unrepaired or improperly repaired, DSBs contribute to chromosomal aberrations, which may lead to human disorders including cancer^(^^16)^. The detectable amount of these DNA damages correlates well with the dose received. However, the biological radiation damage depends not only on dose but also on other individual factors like radiation sensitivity and DNA repair capacity^(^^17)^. In this regard, it is important to not only predict the DSB level arising from a given ionizing radiation (IR) dose, but also to consider the DSB repair rate, which might considerably fluctuate between tissues and individuals^(^^18)^. Until recently, it was impossible to study DSBs production and repair at low irradiation dose due to the very limited sensitivity of the DSB detection methods^(^^18)^. Currently, the potential health risk of CT scan has been discussed in various studies to determine whether low-dose radiation (<100 mSv) could enhance the chromosome aberration yields in pediatric patients. This review summarizes different bioassay systems that are either highly specific for radiation damage (e.g. the dicentric assay) or highly sensitive to low-dose induced DNA damage (e.g. the gamma-H2AX assay) for radiation-exposed children.

## BIOLOGICAL ASSAYS

### The dicentric chromosome assay

The dicentric chromosome assay is currently the most specific method for detecting radiation-induced DNA damage and is regarded as the ‘gold standard’ for biodosimetry^(^^19)^. The dicentric chromosome is an abnormal chromosome with two centromeres. It is an exchange between the centromeric pieces of two broken chromosomes, which in its complete form is accompanied by an acentric fragment (lacking a centromere) composed of the acentric pieces of these chromosomes^(^^20)^. Dicentric aberrations are unstable because their frequency decreases with the turnover of peripheral blood lymphocytes. Thus, for reliable dose assessment, dicentric aberration assays should be performed within a few weeks of exposure. If performed later, the precision of the assay is diminished as the dose calculation requires the use of half-time estimates for the disappearance of dicentric chromosomes^(^^20)^. Earlier data on the chromosome aberrations in peripheral lymphocytes after CT scans have only been obtained in adult patients. Stephan *et al*.^(^^21)^ conducted a small-scale study with samples from ten pediatric patients undergoing CT examination (blood doses in the range of 1.2–31.3 mGy)^(^^21)^. They found that single CT scans significantly elevated dicentric chromosome aberrations in peripheral lymphocytes of children from 0.4–9 years but not from 10 to 15 years of age, indicating that younger children may be more radiosensitive than older subjects, even if the calculated mean blood dose of 16.1 mGy for the children in the younger age group was higher by a factor of 1.7 than the mean dose of 9.7 mGy for the children in the older age group^(^^21)^. Significantly higher levels of dicentric induction were found for the single and combined newborns/children group compared to adults, by a factor of 1.48 (95% confidence interval 1.30–1.68), after *in vitro* exposure to 978 mGy^(^^6)^. For the low dose of 41 mGy, the power of the dicentric assay was not sufficient to detect an age-related radiation sensitivity in the sample size investigated. Precise physical dosimetry was achieved with the *in vitro* approach performed in the study of Gomolka *et al*.^(^^6)^ where an age-dependent sensitivity was found not only in newborns but also in very young children up to five years of age. In addition to the precise physical dosimetry, the strength of this study was in the homogeneity of the examined group, which is focused on healthy children of one gender.

### The micronuclei assay

The micronuclei (MN) assays have emerged as one of the preferred methods for assessing chromosome damage because they enable both chromosome loss and chromosome breakage to be measured reliably^(^^22)^. MN are formed from lagging chromosomal fragments or whole chromosomes at anaphase that are not included in the nuclei of daughter cells during cell division. They are therefore seen as distinctly separate small spherical objects that have the same morphology and staining properties of nuclei, within the cytoplasm of the daughter cells^(^^20)^. Because MN can only be expressed in the cells that complete nuclear division, a special method was developed that identifies such cells by their binucleate appearance when blocked from performing cytokinesis by cytochalasin B, a microfilament-assembly inhibitor. The cytokinesis-block micronucleus assay allows better precision because the data obtained are not confounded by altered cell division kinetics^(^^22)^. The MN assay is a low-cost test for the study of damage of the genome and considered as an intermediate endpoint of carcinogenesis due to radiation exposure (23). A large epidemiological study has shown that the frequency of a micronucleus in peripheral blood lymphocytes can predict the risk of cancer in humans^(^^24)^. Ait-Ali *et al*.^(^^25)^ have demonstrated that median MN values increased significantly after radiological procedures with a median lifetime cumulative effective dose 7.7 mSv per patient (range 4.6–41.2 mSv) in children with congenital heart disease. However, this study has some limitations, like a small number of patients, small contribution of dose originating from CT (11%) and marked variability in the dose of each examination. Khattab and coworkers^(^^14)^ revealed that low-dose CT X-rays are associated with significant biological consequences in precursor cells for erythrocytes in some neonatal children. In infants with no history of a prior CT scan, there was no difference in the average frequencies of micronucleated reticulocytes (MN-RETs) found 2 h before versus 48 h after a scheduled CT scan^(^^14)^. In contrast, in infants who had prior CT scans, the average levels of MN-RETs at 48 h after a scheduled CT scan were significantly higher than their corresponding baseline values, and these increases in MN-RET frequencies were significantly related to the number of previous CT scans. These findings suggest that prior CT scans increase the cellular responses to subsequent CT exposures.

**Table 1:** Overview of published reports about chromosome aberrations and gamma-H2AX foci induced by CT scans

Method	Type of study	No. of patients	Age (year)	Gender (m, f)	Publication	Radiation effect of CT	Exposed dose
Gamma-H2AX assay: manual foci count	*in vivo*	*n* = 3	0.25–1.75	m	Halm *et al*. (31)	Yes	Blood doses of 0.22–1.22 mGy
Gamma-H2AX assay: manual foci count	*in vitro, in vivo*	*n* = 51	0.1–12.2	m, f (37:14)	Vandevoorde *et al*. (33)	Yes	Blood doses of 0.15–8.85 mGy
Dicentric chromosome assay: fluorescence plus Giemsa staining	*in vivo*	*n* = 10	0.42–15	m, f (5:5)	Stephan *et al*. (21)	Yes (only in patients <10 years old)	Blood doses of 1.2–31.3 mGy
Micronucleated reticulocytes: flow cytometry	*in vivo*	*n* = 25	0–1.5	m, f	Khattab *et al*. (14)	Yes (only in infants having prior CT scans)	
Gamma-H2AX assay: automatic, manual foci count and Dicentric chromosome assay: Giemsa staining	*in vitro*	*n* = 15	2–5	m	Gomolka *et al*. (6)	Yes (and increased level of dicentric aberrations only after 978 mGy exposure compared to adults)	Blood doses of 0, 41 and 978 mGy
Micronucleated cytokinesis block assay	*in vivo*	*n* = 59	0–16	m, f (42:17)	Ait-Ali *et al*. (25)	Yes (but CT examination represent only 11% of the total collective dose from various types of medical ionizing procedures)	4.6–41.2 mSv

### The gamma-H2AX assay

The loss of chromosomal integrity from DNA double-strand breaks introduced into mammalian cells by IR results in the specific phosphorylation of histone H2AX on serine residue 139, yielding a specifically modified form named gamma-H2AX. DSB-mediated phosphorylation of H2AX by phosphoinositide 3-kinase-related protein kinases, such ataxia telangiectasia mutated, ATR (ATM- and rad3-related protein) or DNA-dependent protein kinase spreads up to several megabase pairs around the damaged sites and related foci appear within a minute^(^^26)^. The phosphorylation of hundreds to thousands of H2AX molecules from foci at the break sites can be detected by fluorescence microscopy^(^^27)^. The number of gamma-H2AX IR-induced foci, 30 min after irradiation, is similar to the number of DSBs and gamma-H2AX foci disperse once the breaks are repaired^(^^28)^. In some studies, the baseline value of gamma-H2AX foci is achieved again after 24 h after radiation exposure^(^^27^^,^^29)^. Further, due to rapid DSB repair, it is quite possible to underestimate the actual radiation damage, since under some circumstances, the breaks have already been repaired at the time of measurement. To avoid this underestimation, the phosphate inhibitor calyculin A has been employed to prevent the disappearance of gamma-H2AX foci in irradiated cells^(^^30)^. The gamma-H2AX assay is presently used as the most sensitive test system for detecting directly induced radiation damage to DNA, especially in the low-dose range (~1 mGy)^(^^31)^ on a single cell basis^(^^32)^. For example, Halm and coworkers^(^^31)^ found that CT scans involving very low radiation doses (blood doses of 0.22–1.22 mGy) caused a dose-dependent increase in gamma-H2AX foci in T-cells collected one hour after the exam compared to the cells collected before a CT scan (*P* = 0.046). This study has several limitations that need to be emphasized, such as the small number of samples and the absence of any control subjects. Another study of Vandevoorde *et al*.^(^^33)^ provides evidence that CT induces DNA damage in pediatric patients (*n* = 51), at a low dose (blood doses in the range of 0.15–8.85 mGy). The low-dose hypersensitivity observed in the gamma-H2AX foci dose-response of this study indicates that risk estimates based on the LNT (linear-no-threshold) model may potentially underestimate the risks of pediatric CT imaging. The new *in vitro* CT study describes that age-related radiation sensitivity in children was not detected by applying the gamma-H2AX assay^(^^6)^.

Patient-specific factors such as diseases associated with changes to lymphocytes (e.g. infection and lymphoma) or various therapies (e.g. radiotherapy and chemotherapy) can also falsify measurable values. Such patients have been excluded in the most cited studies^(^^17)^. In summary, detailed analysis has suggested that gamma-H2AX analysis has limitations in its utility as a biomarker to detect low dose exposure due to technical and inter-individual variability, by confounding effects caused by exposures other than radiation, including disease states. However, the assay does have the potential to be suitable for the assessment of DSB repair capacity, which is important for assessing the response to radiation exposure^(^^32)^.

## SUMMARY

Several studies have reported the increase of chromosome aberrations in pediatric patients after CT examination *in vitro* and *in vivo* (Table 1.). Nevertheless, the results of most studies showed limitations as it is described above. On the other side, epidemiological studies give contradictory results for post-natal radiation exposure in the low-dose range^(^^6)^; therefore, it is still difficult to draw conclusions about the effects of CT examinations and risk of carcinogenesis. The consideration of appropriate methods with innovative solutions that ultimately help to uncover the effects of low-dose radiation to children and their potential cancer risks need to be evaluated.
